# Evaluation of clinico-radiological, bacteriological, serological, molecular and histological diagnosis of osteoarticular tuberculosis

**DOI:** 10.4103/0019-5413.40253

**Published:** 2008

**Authors:** Anil K Jain, Santosh Kumar Jena, MP Singh, IK Dhammi, VG Ramachadran, Geeta Dev

**Affiliations:** Department of Orthopedics, University College of Medical Sciences and GTB Hospital, New Delhi, India; 1 Department of Microbiology, University College of Medical Sciences and GTB Hospital, New Delhi, India; 2 Department of Pathology, University College of Medical Sciences and GTB Hospital, New Delhi, India

**Keywords:** AFB culture sensitivity, AFB staining, ELISA for tuberculosis, osteoarticular tuberculosis, polymerase chain reaction

## Abstract

**Background:**

The diagnosis of osteoarticular tuberculosis is clinico-radiological in endemic areas. However every patient does not have the classical picture. Osteoarticular tuberculosis is a paucibacillary disease hence bacteriological diagnosis is possible in 10-30% of the cases. The present study is undertaken to correlate clinico-radiological, bacteriological, serological, molecular and histological diagnosis.

**Materials and Methods:**

Fifty clinico-radiologically diagnosed patients of osteoarticular tuberculosis with involvement of dorsal spine (*n* = 35), knee (*n* = 8), shoulder (*n* = 1), elbow (*n* = 2) and lumbar spine lesion (*n* = 4), were analyzed. Tissue was obtained after decompression in 35 cases of dorsal spine and fine needle aspiration in the remaining 15 cases. Tissue obtained was subjected to AFB staining, AFB culture sensitivity, aerobic/anaerobic culture sensitivity histopathological examination and polymerase chain reaction (PCR) using 16srRNA as primer. Serology was performed by ELISA in 27 cases of dorsal spine at admission and one and three months postoperatively.

**Results:**

AFB staining (direct) and AFB culture sensitivity was positive in six (12%) cases. Aerobic/anaerobic culture sensitivity was negative in all cases. Histology was positive for TB in all the cases. The PCR was positive in 49 (98%) cases. All dorsal spine tuberculosis cases showed fall of IgM titer and rise of IgG titer at three months as compared to values at admission.

**Conclusion:**

Histopathology and PCR was diagnostic in all cases of osteoarticular tuberculosis. The serology alone is not diagnostic.

## INTRODUCTION

Osteoarticular tuberculosis involves 2-5% of all tubercular lesions in the body.^1^ Out of which 50% affects the spine.^1^ The diagnosis of osteoarticular tuberculosis and in particular tuberculosis of the spine is clinico-radiological, particularly in the endemic regions. The typical lesion can be diagnosed clinico-radiologically with support of newer imaging modalities like computed tomography/magnetic resonance imaging (CT/MRI); however, tissue diagnosis is a must when there is a slightest doubt. For accurate diagnosis to be established the tubercular bacteria must be recovered from the lesion.^2^ The emerging multidrug resistant strains are posing a threat to cure the tubercular lesion hence the mycobacterium should be isolated and subjected to drug susceptibility test.^2^

Osteoarticular tuberculosis, being a paucibacillary disease and some of the patients are already on antitubercular treatment (ATT) at the time of presentation, hence no single modality like AFB culture sensitivity, AFB staining, histopathology are capable of ascertaining the diagnosis for tuberculosis. Attempts have been made in the past to develop serologic methods for detection of antibodies against mycobacterial antigens.^3^–^5^ These tests may provide a better indication of activity of disease. Newer methods of diagnosis by molecular methods [polymerase chain reaction (PCR)] have been introduced to partly overcome the problems of traditional methods. The results can be obtained within two to three days, thereby helping in early diagnosis and treatment. 6–9 To the best of our knowledge no study has correlated conventional diagnostic methods with the newer diagnostic methods in osteoarticular tuberculosis. The present study is an attempt to evaluate all the diagnostic modalities (AFB smear, AFB culture sensitivity, PCR, histology, serology) to ascertain their efficacy in establishing the diagnosis and treatment.

## MATERIALS AND METHODS

The study was conducted in a tertiary care hospital. Fifty clinico-radiologically diagnosed cases of osteoarticular tuberculosis with involvement of dorsal spine (*n* = 35), knee (*n* = 8), shoulder (*n* = 1), elbow (*n* = 2) and lumbar spine lesion (*n* = 4), were analyzed. Indications for surgery in cases with dorsal spine involvement were deterioration of (*n* = 14) or static (*n* = 13) neural deficit and doubtful diagnosis (*n* = 3). The peripheral limb lesions and lumbar spine cases were aspirated for confirmation of diagnosis. Tissue obtained during surgery and by biopsy was analyzed for (a) direct microscopy and culture of mycobacteria in all cases. Ziehl-Neelsen stain and Lowenstein-Jensen media were used for staining and culture of mycobacteria.^10^ The positive cultures were identified with a set of standard proportional tests for species identification and drug susceptibility testing was performed by proportional method for streptomycin, isoniazid, rifampicin, ethambutol and pyrazinamide. (b) Tissue samples were also analyzed for other aerobic and anaerobic bacteria. Gram's stain and culture sensitivity was used for aerobic bacteria in all cases. Anaerobic culture sensitivity was done in Robertson's cooked meat medium.^10^ (c) Histopathologically the tissue was stained with hematoxylin and eosin stain (*n* = 50) and was seen under microscope for acid-fast bacillus (AFB) and epitheloid cell granuloma, Langherhan's cell with or without caseation.^11^,^12^ (d) Polymerase chain reaction for rapid diagnosis of mycobacterium was done using 16srRNA as a primer (*n* = 50), which is a genus-specific primer, on the granulation tissue/pus obtained. (e) ELISA test was used for serological tests, in all cases. It could be done in only 27 cases of dorsal spine tuberculosis because of paucity of ELISA kits. Serum IgG and IgM levels were measured against 38kd and A-60 tubercular antigen respectively. Blood for serology was taken at the time of admission and then at one and three months postoperatively for serial rise or fall in immunoglobulin titers.

## RESULTS

**Figure 1:**
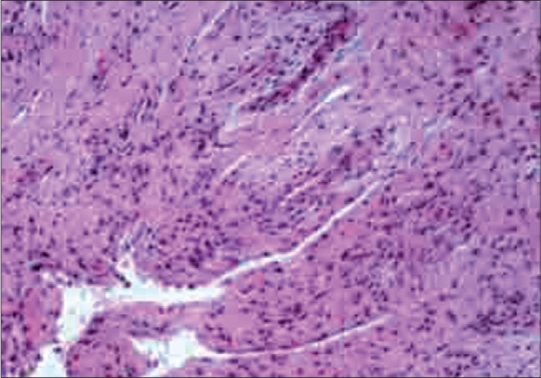
Histopathology slide showing caseation necrosis, epitheloid cell granuloma and Langerhans giant cells

**Figure 2:**
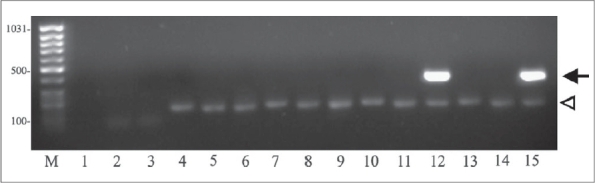
PCR amplification of 16sr RNA of *M. Tuberculosis* in 1.5% agarose gel

The AFB staining (direct) and AFB culture sensitivity was positive in six (12%) cases. These were of the same specimens. All the cases (100%) had histological features suggestive of tubercular osteomyelitis, confirmed by presence of caseation necrosis, epitheloid cell granuloma and Langerhans giant cells [Figure 1]. The PCR was positive for mycobacterium tuberculosis complex in 49 (98%) cases [Figure 2]. All the cases were Gram's stain negative and showed no growth on pus culture sensitivity (Table 1). Mean IgM titer at admission, one month and three months postoperative was 0.9293, 0.6413 and 0.5103. Mean IgG titer at admission, one month and three months postoperative was 0.2974, 0.3027and 0.3341. Serological tests showed fall of IgM and rise of IgG titer at three months as compared to values at admission and at one month post operative (Table 2).

**Table 1:** Results of diagnostic investigations

Investigation	No. of positive cases	Percentages
AFB staining (direct)	6	12
AFB culture	6	12
Histopathology	50	100
PCR	49	98
AFB Staining +	6	12
AFB C/S + Histopathology +		
PCR + Serology		

**Table 2:** Results of ELISA test for *Mycobacterium tuberculosis* complex

Variable	*n*	Mean titer	SD	*P*-value	Significance
IgM					Significant
At admission	27	0.9293	0.5269	0.001	
1-month postop	27	0.6413	0.3077	
3-months postop	27	0.5103	0.2547		
IgG					Significant
At admission	27	0.2974	0.1009	0.001	
1-month postop	27	0.3027	6.6287		
3-months postop	27	0.3341	8.2005		

## DISCUSSION

About 30 million people suffer from tuberculosis (TB) throughout the world every year and 1-2% of these patients suffer from osteoarticular tuberculosis. Diagnosis of osteoarticular tuberculosis is difficult since the organism is fastidious and slow-growing.^2^ AFB is difficult to isolate in osteoarticular tuberculosis since it is a paucibacillary disease and being a deep-seated lesion it is difficult to procure the tissue.^2^

The diagnosis of osteoarticular tuberculosis in endemic areas is clinico-radiological. It is justified to treat the patients clinico-radiologically in classical lesions of the bone. The clinical and radiological response can be observed in 8-12 weeks. However, there are certain cases with doubtful diagnosis, where tissue is required to ascertain diagnosis. In the bone of the appendicular skeleton, tissue may be procured by fine needle aspiration cytology (FNAC) or core biopsy. Delay of a few days in the treatment of the limb lesion does not give rise to severe consequences, as tuberculosis is a slowly progressive disease. Tuberculosis of the spine is a deep-seated lesion, which if not diagnosed promptly and treated adequately, then consequences would be hazardous, as patient may develop kyphosis and/or neurological complication (paraplegia). Osteoarticular tuberculosis, being a paucibacillary disease some of the patients are already on ATT, hence no single modality like AFB culture, AFB staining, histopathology are capable of ascertaining the diagnosis.^2^ The role of serological tests and PCR (molecular methods) are still not well defined in management of osteoarticular tuberculosis.

Sensitivity of AFB staining in various series was reported in the range of 25-75%.^2^ Lakhanpal *et al.*, reported 49.53% positivity by AFB culture sensitivity.^2^ Other workers have reported in a range of 48.6-80%.^2^ In our series AFB staining and AFB culture was positive in six (12%) cases. Out of these six cases four patients were on ATT for more than two months before presentation, one patient was on ATT since last four days and one patient had never taken ATT. In pulmonary TB, since the sputum can be procured easily, direct AFB staining has an important role in its treatment, as the conversion of sputum positive to sputum negative indicates the efficacy of the treatment. However, in osteoarticular tuberculosis, the bacterial diagnosis and absence of mycobacterium tuberculosis on treatment cannot be taken as a criterion of successful treatment. Lakhanpal *et al.*,^2^ attributed the lower percentage to the possible effect of preoperative antitubercular therapy. Wilkinson also asserted the same point. Our positivity of AFB staining was very low in spite of 16 (32%) of our cases having never taken ATT before reporting to the hospital. However, status of taking other broad-spectrum antibiotics is not known, which may have an inhibitory effect on mycobacteria. Therefore, the fact that duration of ATT has any influence on the positivity of AFB culture sensitivity could not be substantiated in our study. Factors attributable for less positivity with AFB culture sensitivity are paucibacillary disease (Number of AFB is about 103-104/ml), species present (*M. tuberculosis* is more likely to be positive than MOTT), patient already on ATT, stain used, observer's experience. The major limitations of AFB culture and sensitivity are that it requires live organisms, has a long incubation period, and low sensitivity in patients already on ATT. Although the culture results are not available for up to four weeks, they prove the diagnosis of TB beyond doubt.^2^ Newer rapid culture techniques for diagnosis of TB like BACTEC and BACTEC-alert would be better alternatives compared to conventional methods.

Due to the need of avoiding culture techniques, which carry the hazard of handling live organisms, various serological methods have been developed to detect serum antibodies against *M. tuberculosis*. Various authors have tested various antigens, which could be specific for *M. tuberculosis*.^4^,^5^ Serological tests showed fall of IgM and rise of IgG titer at three months postoperatively as compared to values at admission and at one month postoperatively. There was significant difference in the values of IgM and IgG at the time of admission and at three months postoperatively. These antibodies' titers did not correlate with the recovery status of the patient, as patients did not show recovery proportionally to the declination of IgM titer. IgM titer was diagnostic of activity of the disease while IgG titer was diagnostic of chronic disease. The IgG levels remain high even after full treatment.^4^,^5^ Although IgG levels have no diagnostic value, they suggest that the patient has chronic disease or healed disease. Serological tests' results did not correlate with the duration of ATT intake. This means that ELISA values are dependent on time of taking sample and state or phase of disease. Whether patient is smear-negative or has pulmonary or extrapulmonary TB cannot be distinguished by this method. These observations on serological test results are essentially the same as those in other series.^4^,^5^

A large number of organisms required by all the above methods was the major limitation in detection of *M. tuberculosis*. A single test, which would amplify the genome, even if a single organism was present, was thought to be ideal for detection of paucibacillary TB cases. The PCR can analyze the expression of genes even from single cells. The PCR was positive in 49 (98%) cases in our series. One case who was PCR-negative had a histopathology report suggestive of tubercular osteomyelitis. Our results with PCR were comparable with other authors' series.^6^,^7^,^8^,^13^–^16^ A number of target genes of mycobacteria DNA have been evaluated for diagnosis by PCR. In the present study, we have used 16srRNA as target sequence as it is universally present and hence rules out the chances of false negative results. It is a genus-specific marker and present in both typical and atypical mycobacteria. Combination of both PCR [IS6110 and 16srRNA{multiplex}] is preferred.

*The advantages of PCR are*: (1). It is a highly efficient and rapid method for diagnosis of the disease [24h]. (2). A PCR result is of great value in early diagnosis, particularly in infection of certain body systems where disease progression is very fast and detection by culture method is time-consuming. (3). As PCR is a very sensitive technique and could detect as few as one to two mycobacteria in the specimen, treatment may be initiated based on this result, if there are clinical signs of the disease (4). It can differentiate typical and atypical mycobacteria. (5) A PCR requires a very small quantity of specimen and therefore, even microliters of a fine needle aspirate can be tested. A PCR can detect very low levels of AFB in the clinical specimens and results are available within three days. However, misleading results can occur as even the smallest amount of contaminating DNA can be amplified. A PCR-positive result does not always confirm to culture results. A PCR is not a substitute for culture; it is an addition to the routine battery of laboratory tests for the rapid and definitive diagnosis of tuberculosis.

*The disadvantages of PCR are*: It is not able to differentiate live from dead organisms as it is not dependent on bacterial replication. It does not tell about the activity of the disease.

Histopathological diagnosis of osteoarticular TB has been reported in the range of 72-97%.^2^,^17^ Histopathological positivity in our series was 100%. All the cases had histological features suggestive of tubercular osteomyelitis, confirmed by presence of caseation necrosis, epithelioid cell granuloma and Langerhans giant cells. In our series positivity with histopathology was more as compared to other series.^2^,^17^ Sometimes the results were inconclusive as the patients were already on ATT for many months before reporting to the hospital. The advantage of histopathology lies in the early results obtained, enabling the surgeon to embark upon the appropriate treatment. Silverman *et al.*, stated that fine needle aspiration (FNA) biopsy could provide diagnostic material for morphologic and microbiologic confirmation, while avoiding either core biopsies or open biopsies under general anesthesia. With the cytomorphologic recognition of a granulomatous process presumptive diagnosis of skeletal TB can be made, thus expediting early appropriate antitubercular treatment and excluding other processes.^18^ Mondal A stated that CT-guided fine needle aspiration cytology (FNAC) is a useful and minimally invasive method of ascertaining histopathological diagnosis of vertebral lesions.^19^ In his series all 38 cases had histopathological features of skeletal tuberculosis.

Combined efficacy of histopathology, culture sensitivity and guinea pig inoculation as reported by Lakhanpal *et al.*, had 100% positive results.^2^ Saxena *et al.*, reported the positivity of combined efficacy as 76.4%.^17^ Van der Spoel van Dijk *et al.*, reported that detection using culture could confirm only three (11.53%) of the 26 clinically diagnosed TB cases while PCR detection confirmed disease in 15 (57.69%) cases.

Calculated sensitivity and specificity of PCR employing culture as the “gold standard” were 100% (with 95% CI 29.2; 100.0) and 71.4% (55.4; 84.3), which due to low detection levels, basically excludes culture as a standard for statistical analysis. Sensitivity and specificity of PCR using histology as the “gold standard” were 78.6% (49.2; 95.3) and 87.1% (70.2; 96.4) respectively with positive and negative predictive values of 73.3% (44.9; 92.2) and 90% (73.5; 97.9) respectively.^14^ Titov *et al.*, stated that conventional bacteriological methods for demonstration of *M. tuberculosis* are not very sensitive and can be time-consuming. The PCR of arthroscopically obtained joint tissue biopsies appears promising in the early diagnosis of tuberculous arthritis.^20^ Only six (12%) cases of dorsal spine tuberculosis were positive for combined PCR, histopathology, AFB staining and AFB culture sensitivity and serological tests in our series.

We conclude that high sensitivity and specificity of PCR and histopathology can be useful for early diagnosis of paucibacillary osteoarticular tuberculosis. However, once a tissue is procured it should be subjected to AFB staining, AFB culture sensitivity, PCR and histopathology in all the cases.
